# Carbon Monoxide Modulates Connexin Function through a Lipid Peroxidation-Dependent Process: A Hypothesis

**DOI:** 10.3389/fphys.2016.00259

**Published:** 2016-06-28

**Authors:** Mauricio A. Retamal

**Affiliations:** Centro de Fisiología Celular e Integrativa, Facultad de Medicina, Clínica Alemana Universidad del DesarrolloSantiago, Chile

**Keywords:** hemichannels, connexins, carbon monoxide, lipid peroxides, PUFAs

## Abstract

Hemichannels are ion channels composed of six connexins (Cxs), and they have the peculiarity to be permeable not only to ions, but also to molecules such as ATP and glutamate. Under physiological conditions they present a low open probability, which is sufficient to enable them to participate in several physiological functions. However, massive and/or prolonged hemichannel opening induces or accelerates cell death. Therefore, the study of the molecular mechanisms that control hemichannel activity appears to be essential for understanding several physiological and pathological processes. Carbon monoxide (CO) is a gaseous transmitter that modulates many cellular processes, some of them through modulation of ion channel activity. CO exerts its biological actions through the activation of guanylate cyclase and/or inducing direct carbonylation of proline, threonine, lysine, and arginine. It is well accepted that guanylate cyclase dependent pathway and direct carbonylation, are not sensitive to reducing agents. However, it is important to point out that CO—through a lipid peroxide dependent process—can also induce a secondary carbonylation in cysteine groups, which is sensitive to reducing agents. Recently, in our laboratory we demonstrated that the application of CO donors to the bath solution inhibited Cx46 hemichannel currents in *Xenopus laevis* oocytes, a phenomenon that was fully reverted by reducing agents. Therefore, a plausible mechanism of CO-induced Cx46 hemichannel inhibition is through Cx46-lipid oxidation. In this work, I will present current evidence and some preliminary results that support the following hypothesis: Carbon monoxide inhibits Cx46 HCs through a lipid peroxidation-dependent process. The main goal of this paper is to broaden the scientific community interest in studying the relationship between CO-Fatty acids and hemichannels, which will pave the way to more research directed to the understanding of the molecular mechanism(s) that control the opening and closing of hemichannels in both physiological and pathological conditions.

## Introduction

**Figure 1:**
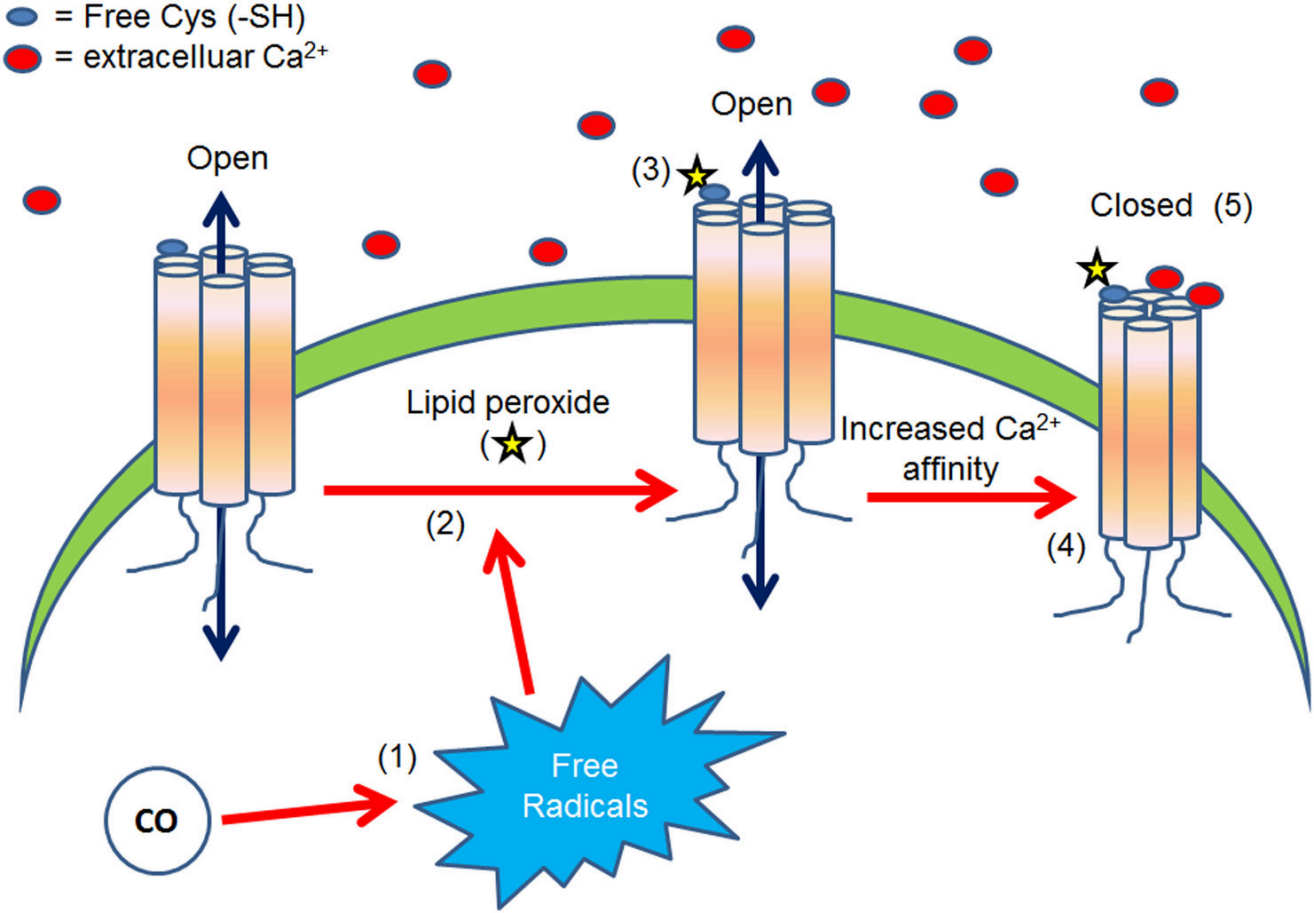
**The above diagram presents what is proposed as a possible molecular mechanism to explain the CO-induced Cx46 inhibition**. CO increases free radicals concentration (1) which in turn induces lipid peroxide production (2). Then, lipid peroxides can induce the carbonylation of extracellular cysteine (-SH) (3), increasing Cx46-Ca^2+^ sensitivity (4) and thus stabilizing the closed state of the loop gating (5).

The hypothesis of the present work is: Carbon monoxide modulates connexin function through a lipid peroxidation-dependent process (Figure 1). This hypothesis is supported by the following knowledge and preliminary data.

## General characteristics of connexins

Connexins (Cxs) are transmembrane proteins that share a common topology: four transmembrane domains, two extracellular loops, one intracellular loop and the C, and N-termini located both on the cytoplasmic side. Twenty isoforms have been described in mammals (Reviewed in Eiberger et al., 2001), which are named following their expected molecular weight (e.g., Cx26 is expected to have an MW of ~26 kDa). Even when Cx isoforms exhibit considerable homology, the C-terminus is the most variable region which differs in length and number of regulatory sites, which include consensus phosphorylation (Reviewed in Lampe and Lau, 2000), protein-protein interaction (Flores et al., 2008; Reviewed in Hervé et al., 2012) and cleavage sites (Lin et al., 1997). Almost all Cxs (except for Cx23; Lovine et al., 2008) have six conserved extracellular-loop cysteines (Cys), which have been proposed are essential for gap-junction channel (GJC) formation (Dahl et al., 1991). Finally, it is important to note that in mammals, virtually all cell types express at least one Cx type, but there are major differences in expression at the tissue level. Thus, for example, Cx43 is the most ubiquitously expressed (Beyer et al., 1987), whereas Cx46 has been described mostly in the lens (Paul et al., 1991). The widespread expression of Cxs suggests that they are essential for several physiological processes and that, due to their unique properties, they support cellular processes that cannot be replaced by any other Cx type (Wölfle et al., 2007).

## Connexin hemichannels

Hemichannels are composed of six Cxs monomers and are permeable to molecules up to ~1.2 kDa. These ion channels participate in several physiological functions, such as spreading of calcium waves (Cotrina et al., 1998), Ca^2+^ permeation across plasma membrane (Sánchez et al., 2010; Schalper et al., 2010), cellular viability (Bellido and Plotkin, 2010), proliferation (Song et al., 2010), migration (Cotrina et al., 2008), light processing by the retina (Kamermans et al., 2001), mechanotransduction (Romanello et al., 2003), glucose uptake (Retamal et al., 2007), and synaptic plasticity (Stehberg et al., 2012), among others. Most of the hemichannel actions are exerted in part by the release of signaling molecules such as ATP (Stout et al., 2002), cyclic ADP-ribose (cADPR) (Bruzzone et al., 2001), prostaglandin E2 (PGE_2_) (Cherian et al., 2005), glutamate, and aspartate (Ye et al., 2003) to the extracellular media, where they participate in paracrine/autocrine communication. On the other hand, under pathological conditions, massive and/or prolonged hemichannel opening induce and/or accelerate cell death. Nowadays, hemichannels with a gain of activity are known as “leaky hemichannels,” and these have been observed in neurological disorders such as Charcot-Marie-Tooth disease, metabolic alterations such as ischemia, oculodentodigital dysplasia, skin diseases, inflammatory processes, and deafness (Reviewed in Retamal et al., 2015a). Although the mechanism by which hemichannels induce cell death is not well understood, it is highly probable that loss of metabolites (Stridh et al., 2008), ion gradients and membrane potential, as well as the massive entry of Ca^2+^ (Sánchez et al., 2010; Schalper et al., 2010) are some of the processes involved. Because hemichannels are important in cellular communication and cell survival, cells have several mechanisms to control hemichannel opening/closing, including phosphorylation (Bao et al., 2004), changes in membrane potential (Trexler et al., 1996; Retamal et al., 2010), alterations in extracellular Ca^2+^ concentration (Gómez-Hernández et al., 2003; Lopez et al., 2014), changes in redox potential (Retamal et al., 2006, 2009; Reviewed in Retamal, 2014) and presence of unsaturated fatty acids (Retamal et al., 2011). In summary, controlled hemichannel opening enables physiological autocrine/paracrine cell communication, but massive and/or uncontrolled hemichannel opening induces or accelerates cell death. Therefore, the study of the molecular mechanisms that control hemichannel activity is essential in order to understand several physiological and pathological processes.

## Hemichannel voltage gating

Currently, it is well accepted that Cx channels are voltage dependent (Trexler et al., 1996; Retamal et al., 2010), although the molecular mechanisms that regulates the hemichannel voltage gating are not yet well understood. But, recently it was established by means of exchange different domains between Cx46 and Cx50 that the N-terminus contains the principal components of the hemichannel voltage sensor and unitary conductance (Kronengold et al., 2012). Additionally, there are two gating mechanisms that enable hemichannels to open and close, which are known as fast and slow/loop-gating (Reviewed in Oh and Bargiello, 2015). The first seems to depend on C-terminus docking to the intracellular loop and results in fast transitions from the open state to various substates (Reviewed in Oh and Bargiello, 2015). In contrast, slow or loop-gating is formed by the interface between the first transmembrane domain (TM1) and the first extracellular loop (EL1; Tang et al., 2009). Their distinctive feature is the slow time constant from fully open to fully closed states (tens to hundreds of milliseconds; Reviewed in Oh and Bargiello, 2015). Large conformational changes reduce the pore diameter from ~20 Å to less than 4 Å (Verselis et al., 2009). It is widely accepted that extracellular divalent cations reduce the open probability of hemichannels in the plasma membrane (Gómez-Hernández et al., 2003; Lopez et al., 2014). Physiological concentrations of Ca^2+^ have been shown to stabilize the loop-gate closed state of Cx46 hemichannels (Verselis et al., 2009; Reviewed in Oh and Bargiello, 2015). Furthermore, atomic force microscopy studies of Cx26 hemichannels have indicated a narrowing of the extracellular channel entrance with 2 mM Ca^2+^ (Müller et al., 2002).

## Carbon monoxide

There are at least four gaseous transmitters, carbon monoxide (CO), nitric oxide (NO), hydrogen sulfide (H_2_S; Reviewed in Farrugia and Szurszewski, 2014), and sulfur dioxide (SO_2_; Chen et al., 2015), which are important modulators of the redox status and redox signaling. Under physiological conditions, CO is produced by two heme oxygenases (HO-1 and HO-2), which catalyze the catabolism of heme groups (Poss and Tonegawa, 1997). Both HO-1 and HO-2 are expressed in several cell types, and while HO-2 is constitutively expressed, HO-1 is inducible by several factors such as hypoxia and inflammation (Reviewed in Wu and Wang, 2005). Under physiological conditions, the human body produces 16.4 μmoles/h (Coburn, 1970), mainly by the action of HO-2 (Reviewed in Wu and Wang, 2005). Once CO is produced, it can be trapped by the hemoglobin, released by expiration (Reviewed in Wu and Wang, 2005) or act as a signaling molecule. In spite of the low concentration (nanomolar) of CO under physiological conditions, it has important roles in normal cardiac function, vascular contractility, platelet aggregation, monocyte activation, hypothalamic-pituitary-adrenal axis, odor response adaptation, nociception and chemoreception, among many other functions (for more details see, Wu and Wang, 2005). Additionally, CO production under physiological conditions can be increased by the induction of HO-1 controlled by physiological signaling molecules, such as transforming growth factor-β (Kutty et al., 1994), platelet-derived growth factor (Durante et al., 1999), and nitric oxide (Durante et al., 1997). Or, it can be decreased by angiotensin II (Ishizaka and Griendling, 1997). On the other hand, under pathological conditions HO-1 can be highly expressed and thus drastically increasing the CO levels (Reviewed in Wu and Wang, 2005). The expression of HO-1 has been implicated in diseases such as atherosclerosis, hypertension, transplant rejection, acute renal injury hyperoxia and hypoxia-induced lung injury, cancer, and neurodegeneration, among others diseases (Deshane et al., 2005; Reviewed in Wu and Wang, 2005). Although CO does not have free electrons as nitric oxide does, it can indirectly increase the oxidative stage of a cell. Thus, at high levels (>1000 ppm), CO increases protein and lipid oxidation (Reviewed in Wu and Wang, 2005), most likely as a result nitric oxide derived molecule production and dysregulation of GSH/GSSG relationship (Reviewed in Wu and Wang, 2005).

CO can act as a signaling molecule through two possible cellular pathways. First, the direct activation of guanylate cyclase, increases cGMP levels, which in turn activate PKG; and secondly, CO acts by direct carbonylation of amino acids, such as proline, threonine, lysine, and arginine (Reviewed in Cattaruzza and Hecker, 2008). However, CO can also induce an indirect carbonylation of cysteine residues through a lipid peroxidation dependent process (Reviewed in Wong et al., 2013). Thom (1990) showed that CO-dependent lipid peroxidation is reduced by the inhibition of xanthine oxidase or superoxide dismutase and iron chelators. Additionally, high concentration of CO was associated with increases in hydroxyl radical production and decreases in the reduced -oxidized glutathione ratio (GSH/GSSG; Lautier et al., 1992; Piantadosi et al., 1995; Reviewed in Wu and Wang, 2005). Therefore, CO can induce an oxidative intracellular environment, which in turn can favor the lipid peroxidation production rate. The process of lipid peroxidation is mediated through both enzymatic and non-enzymatic oxidation of poly unsaturated fatty acids (PUFAs; Reviewed in Higdon et al., 2012). Enzymatic sources of lipid peroxides comprise both COXs (cyclo-oxygenases) that produce PG (prostaglandins) and LOXs (lipoxygenases) that produce leukotrienes (Reviewed in Higdon et al., 2012). On the other hand, non-enzymatic production is mediated by direct oxidation of PUFAs and comprises the production of 4-HNE (4-hydroxynonenal), malondialdehyde (MDA), and acrolein (Reviewed in Higdon et al., 2012). Interestingly, the secondary carbonylation of the Toll-like receptor by 4-HNE can be prevented by reducing agents (Kim et al., 2009), demonstrating that lipid peroxide-induced carbonylation is a dynamic process that can be modulated by the cellular redox status.

## Carbon monoxide modulates ion channels

In the early nineteens was described that mice exposure to high levels of CO-gas present degeneration of hippocampal CA1 pyramidal cells by a NMDA-dependent process, measured with hematoxylin-eosin staining (Ishimaru et al., 1992). This suggests that CO may induce neuronal cell death through changes of ion-channel activity. From this work, several reports strongly supported the notion that CO acts as an ion-channel modulator. Thus, it has been shown that CO increases the open probability of calcium-activated K^+^ (KCa) channels in vascular smooth muscle cells (Wang et al., 1997) and human umbilical vein endothelial cells (Dong et al., 2007). The molecular mechanism of this phenomenon is not well understood, but it has been proposed that depends on the increase of the number of Ca^2+^ binding sites (Wang et al., 1997), expression of the alpha subunit (Wu et al., 2002), modulation by NO (Wang and Wu, 2003), and a metal-dependent like coordination of CO by Cys at position 911 (C911) (Williams et al., 2008; Telezhkin et al., 2011). Other ion channels are also affected by CO, such as a 70-pS K^+^ channel in the thick ascending limb of Henle's loop (Liu et al., 1999), Kv2.1 (Dallas et al., 2011), hTREK-1 (Dallas et al., 2008), the amiloride-sensitive Na^+^ channel (Althaus et al., 2009), Nav1.5 channels (Elies et al., 2014), Cav3.2 (Boycott et al., 2013), and P2X2 receptors (Wilkinson et al., 2009). In the case of Cav3.2 channels, CO induced-inhibition was dependent on the activation of an extracellular thioredoxin-dependent mechanism (Scragg et al., 2008). Together, these data suggest that CO exerts many of its effects through ion-channel modulation.

## Carbon monoxide modulates Cx-hemichannels

Recently, was demonstrated that CO is a new hemichannel modulator (León-Paravic et al., 2014; Reviewed in Retamal et al., 2015b). The application of CO donors (CORM-A1, CORM-2, and CORM-3) to the bath solution inhibited the currents of Cx46 hemichannels expressed in *Xenopus laevis* oocytes (*X*. oocytes). The inhibition has an IC_50_ of approximated 3.4 μM, making Cx46 hemichannels an excellent CO sensor under pathological (>10 μM) condition (Kajimura et al., 2010). Moreover, CORM-2 effect was fully prevented by the addition of hemoglobin (a CO scavenger) to the bath solution and was correlated with Cx46 carbonylation, which in turn, produced important protein structural rearrangements *in vitro* (León-Paravic et al., 2014). Interestingly, the effect of CO did not involve changes in voltage dependency or modifications of the C-terminus. Additionally, hemichannels formed by Cx46 lacking extracellular-loop Cys were much less sensitive to CORM-2 compared to wild type Cx46 hemichannels. Moreover, hemichannel inhibition was fully recovered by addition of reducing agents to the bath solution (e.g., GSH and DTT; León-Paravic et al., 2014). The extracellular cysteine redox status potentially could affect the conformational disposition of the loop-gating, which in turn, is known to affect the loop- gating (Reviewed in Retamal et al., 2016). From these data it can be proposed that CO could inhibit Cx46 hemichannels through changes of the loop-gating properties, likely enhancing the effect of Ca^2+^ (Figure 1).

For many years, protein carbonylation was considered synonymous with protein degradation (Reviewed in Wong et al., 2013). However, recent evidence suggests that there is a naturally occurring process of protein decarbonylation (Wong et al., 2008; Reviewed in Wong et al., 2013). This mechanism involves an unknown thiol-dependent enzymatic process, in which the enzymes thioredoxin (Trx) and glutaredoxin (Grx1) seem to play important roles (Wong et al., 2008; Reviewed in Wong et al., 2013). Therefore, based on the current knowledge, the effect of CO (most likely secondary carbonylation) upon protein activity can be reversed and controlled by the redox status of a cell. Nevertheless, the exact molecular mechanism of decarbonylation is poorly understood. In our study (León-Paravic et al., 2014) we blocked TRx with aurarofin and a small recovery of hemichannel current was observed, which suggests that TRx does not play an important role in the recovery of hemichannel current induced by reducing agents. The question still remains as to whether GRx could participate. As indicated, CO can also act indirectly through lipid peroxidation of Cys groups (Reviewed in Wong et al., 2013; Milic et al., 2015). Lipid-induced protein oxidation can be reverted by glutathione peroxidase (GPx4) and glutathione S-transferase (GST; Reviewed in Ribas et al., 2014), which are activated by reducing agents (Reviewed in Ribas et al., 2014). Therefore, a plausible mechanism of CO-induced Cx46 hemichannel inhibition is through Cx46-lipid oxidation.

**Figure 2:**
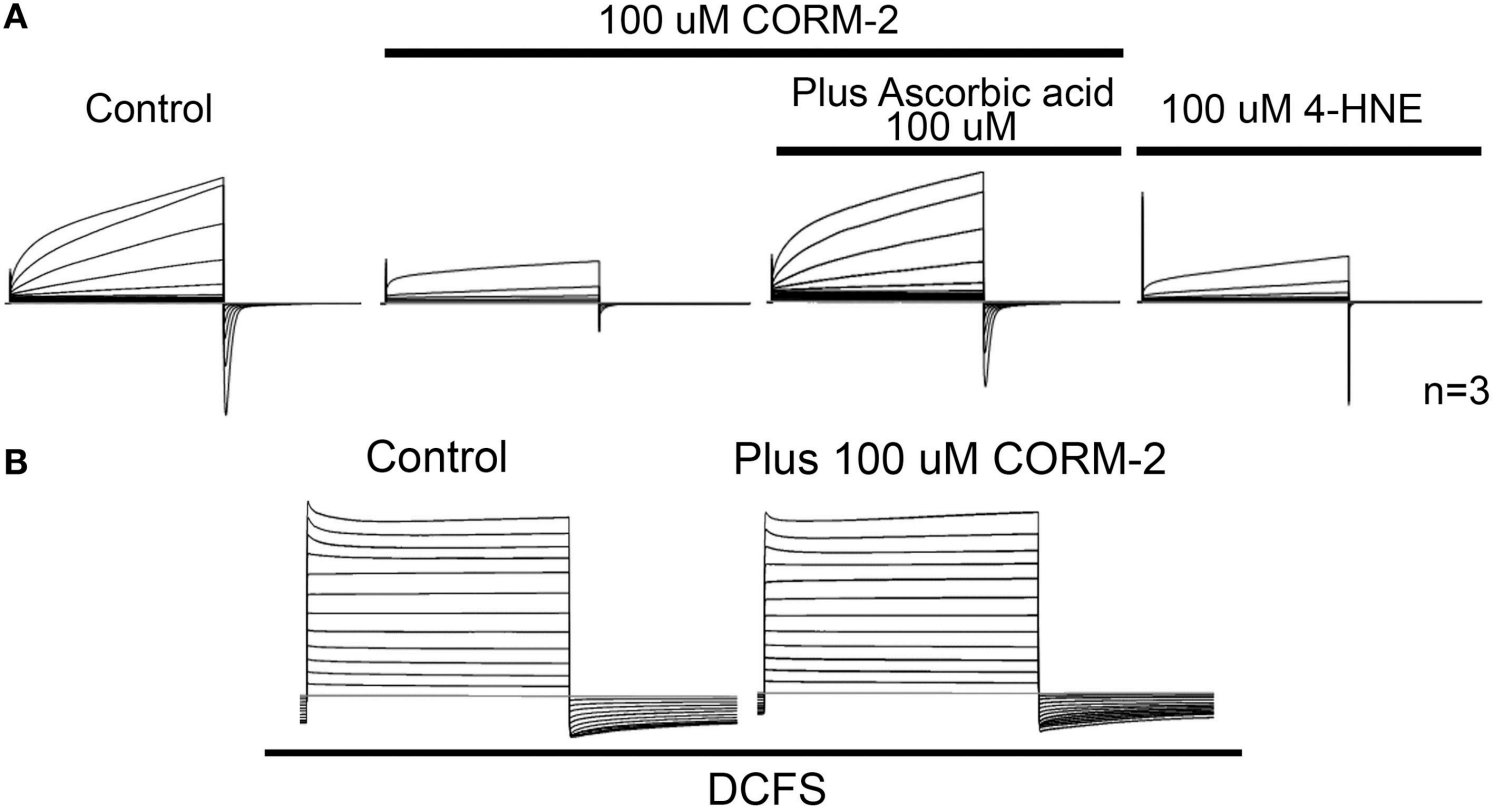
**CO effect appears to be mediated by lipid peroxides. (A)** Representative control of Cx46 hemichannel currents in *Xenopus laevis* oocytes recorded in ND96 solution (containing 1.8 mM Ca^2+^ and 1.0 mM Mg^2+^) by means of dual whole cell voltage clamp technique. The presence of 100 μM CORM-2 induces a dramatic drop in the current amplitude. Most of the inhibition induced by CORM-2 was prevented by the co-addition of 100 μM ascorbic acid to the bath solution. This suggests that the effect of CO needs free radical production into the *Xenopus* oocytes. In parallel experiments, oocytes expressing Cx46 were exposed to the lipid peroxide 4-HNE (100 μM), and an evident hemichannel current inhibition was observed. *n* = 3 for each condition. **(B)** Representative recordings of oocytes expressing Cx46 placed in a DCFS, without (control) or with 100 μM CORM-2 (*n* = 3).

In support of the role of oxidized lipids on Cx46 hemichannel inhibition, polyunsaturated fatty acids (PUFAs), such as linoleic acid and arachidonic acids, which can be easily oxidized (Reviewed in Ribas et al., 2014), inhibits Cx46 hemichannels *in vitro* (Retamal et al., 2011). Therefore, it cannot be ruled out that PUFAs may exert their inhibitory effects upon hemichannels through their oxidized-derived molecules. Moreover, preliminary results have shown that 4-Hydroxy-2-nonenal (4-HNE), a reactive aldehyde derived from oxidized lipids (100 μM), inhibits by 60 ± 12% Cx46 hemichannels in *X*. oocytes (Figure 2) and Vitamin C—a lipid peroxide inhibitor—reduced by ~50% the effect of CO upon Cx46 hemichannels (Figure 2). Moreover, the presence of Ca^2+^ in the extracellular media is fundamental for observing CO-induced inhibition of Cx46 hemichannels, suggesting that the CO-induced inhibition/extracellular Cys-lipid peroxidation involve certain conformational changes that alter loop gating properties (Figure 2).

## Future directions

Hemichannels are relevant players in the development and progression of several diseases, and they are now used as targets for developing new molecules for disease treatments (Reviewed in Retamal et al., 2015a). However, in spite of years of research, the molecular mechanisms that control the opening and closing of these channels are still not well understood. Thus, it is highly relevant to understand these mechanisms and project this knowledge to produce new agonist(s)/antagonist(s) against Cx- hemichannels, as well as to understand why hemichannels become lethal under certain pathological conditions. Although the effect of CO upon GJC has not been studied, it is possible to propose that CO may not have a relevant impact in GJC properties. It because, CO/lipid peroxides seem to act through modifications of extracellular Cys, which in GJC are not accessible for modifications by reducing nor oxidant molecules.

CO has been used for the treatment of several diseases, but many of its effects are far from being well understood. Therefore, the current knowledge is insufficient for understanding how CO exerts its action at the cellular level and, thus, to find possible side effects of this treatment. Also, this knowledge may help to develop new strategies in the therapeutic use of CO, e.g., under metabolic stress, where hemichannels become massively open, which accelerates cell death. Therefore, a possible application of this research would be to pursue the use of CO as a hemichannel inhibitor in preventing or limiting stroke-induced cell death.

## Author contributions

MR wrote this paper and did all the figures.

### Conflict of interest statement

The author declares that the research was conducted in the absence of any commercial or financial relationships that could be construed as a potential conflict of interest.
